# Maternal-foetal complications in pregnancy: a retrospective comparison between type 1 and type 2 diabetes mellitus

**DOI:** 10.1186/s12884-021-03702-y

**Published:** 2021-03-22

**Authors:** Valentina Guarnotta, Mariagrazia Irene Mineo, Emanuela Giacchetto, Maria Pia Imbergamo, Carla Giordano

**Affiliations:** Dipartimento di Promozione della Salute, Materno-Infantile, Medicina Interna e Specialistica di Eccellenza “G. D’Alessandro” (PROMISE), Sezione di Malattie Endocrine, del Ricambio e della Nutrizione, Università di Palermo, Piazza delle Cliniche 2, 90127 Palermo, Italy

**Keywords:** Abortion, Large for gestational age, Macrosomia, Insulin requirement

## Abstract

**Background:**

The aim of the study was a retrospective comparison of the differences in maternal-foetal outcomes between women with type 1 and type 2 diabetes mellitus (T1DM and T2DM).

**Methods:**

A cohort of 135 patients with pregestational diabetes, 73 with T1DM (mean age 29 ± 5 years) and 62 with T2DM (mean age 33 ± 6 years), in intensive insulin treatment throughout pregnancy were evaluated. Clinical and metabolic parameters and the prevalence of maternal and foetal complications were assessed.

**Results:**

Women with T1DM showed lower pregestational BMI (*p* < 0.001), pregestational weight (*p* < 0.001), weight at delivery (*p* < 0.001), ∆_total_insulin requirement (IR) at the first, second and third trimesters (all *p* < 0.001) and higher weight gain during pregnancy (*p* < 0.001), pregestational HbA1c (*p* = 0.040), HbA1c in the first (*p* = 0.004), second (*p* = 0.020) and third (*p* = 0.010) trimesters compared to T2DM. Women with T1DM had a higher risk of macrosomia (*p* = 0.005) than T2DM, while women with T2DM showed higher prevalence of abortion (*p* = 0.037) than T1DM. At multivariate analysis, pregestational BMI and ∆_total_IR of the first trimester were independently associated with abortion in T2DM, while weight gain during pregnancy was independently associated with macrosomia in T1DM.

**Conclusion:**

Women with T1DM have a higher risk of macrosomia than T2DM due to weight gain throughout pregnancy. By contrast, women with T2DM have a higher risk of spontaneous abortion than T1DM, due to pregestational BMI and ∆_total_IR in the first trimester.

## Background

The prevalence of pregestational diabetes among women in the reproductive age is increasing. From 5 to 16% of the pregnant population have gestational diabetes and about 1% have pregestational type 1 diabetes mellitus (T1DM) and type 2 diabetes mellitus (T2DM) [1].

Pregnancies complicated by diabetes mellitus are currently still characterized by a high incidence of unfavourable maternal and foetal outcomes, probably related to poor glycaemic control, particularly in the periconceptional period and in the first trimester of pregnancy [2]. Generally, the perinatal/neonatal specific risks of diabetes in pregnancy include spontaneous abortion (before the 24th week), foetal abnormalities, preeclampsia, perinatal death, macrosomia (>97th percentile), neonatal hypoglycaemia, hyperbilirubinemia and neonatal respiratory distress syndrome. In addition, women with pregestational T1DM may have an aggravation of diabetes microvascular complications such as retinopathy and nephropathy, while those with pregestational T2DM may have a worsening of pre-existing chronic hypertension and obesity [3–5].

Pregnancy is physiologically characterized by reduced insulin sensitivity due to the effects caused by placental hormones, such as human placental lactogen, progesterone, prolactin, placental growth hormone, and cortisol. This change in maternal metabolism is directed towards providing adequate nutrition for the foetus [5, 6].

Although it is known that diabetes mellitus transforms pregnancy into high risk pregnancy [5–7], a controversy still exists on which factors are associated with adverse perinatal outcomes in women with T1DM and T2DM.

The aim of this study was the comparison of maternal demographic characteristics, glycaemic control and pregnancy outcomes between cohorts of T1DM and T2DM patients.

## Methods

We conducted a real-life retrospective cohort study on 135 pregnant women with pregestational diabetes (73 with T1DM, and 62 with T2DM on basal bolus insulin regimen), followed at the Unit of Endocrinology, University of Palermo (Italy) from June 2012 to June 2017. Diagnosis of T1DM and T2DM was made according to the ADA guidelines [4]. All procedures in the study were in accordance with the ethical standards of the local committee on human experimentation (institutional and national) and with the Declaration of Helsinki (1964), as revised in 2013. Approval was obtained from the Ethics Committee of the Policlinico Paolo Giaccone Hospital, University of Palermo. At the time of the first visit in our Out-Patients’ Clinic, all patients included in the study gave written informed consent for the scientific use of their data. Inclusion criteria were: duration of diabetes mellitus of at least 1 year before pregnancy, basal bolus insulin treatment for T1DM and oral hypoglycaemic drugs treatment for T2DM. Exclusion criteria were: twin pregnancies, T1DM on insulin therapy by continuous subcutaneous insulin infusion and T2DM on diet treatment.

Conventional basal bolus insulin therapy consisted of a minimum of 4 daily subcutaneous insulin doses, 3 of a short-acting analogue before the main meals and 1 of a long-acting analogue after dinner.

During the first visit between the 5th and 8th weeks of amenorrhea, a detailed medical history was extracted for each patient, with particular attention to the previous obstetric history, diabetic disease and related chronic complications (retinopathy, nephropathy and arterial hypertension), history of recurrent miscarriages, duration of diabetes and age at pregnancy. Body mass index (BMI) and weight were extracted from the charts. All patients had been trained in self-monitoring of blood glucose (SMBG) using a glucose meter, with the instruction to perform a minimum of 6 daily measurements (before and 2 h after meals), in order to modify and optimize insulin therapy, if necessary. The following target glucose levels were recommended: fasting glucose levels between 3.9 and 5 mmol/L and glucose levels 2 h after the meal of less than 6.7 mmol/L. Patients also received a diet plan based on their pregestational BMI and weight. Patients with pregestational BMI ≥30 kg/m^2^ were considered obese. Hypoglycaemia was defined as glucose level < 3.9 mmol/L. At early gestation and after 4 weeks, glycated hemoglobin (HbA1c) was assessed and repeated every 3 months.

The outpatient visits were at intervals of 2 or 3 weeks until delivery, after which a re-evaluation was carried out after 30 days and 3–4 months after delivery. Acute complications were recorded during follow-up: episodes of ketosis, ketoacidosis and hypoglycaemic events. Mean fasting plasma glucose (FPG), postprandial breakfast glucose (PBG), postprandial lunch glucose (PLG) and postprandial dinner glucose (PDG) at the first, second and third trimesters of pregnancy were recorded for each patient.

Maternal outcomes were also assessed. Nephropathy was defined as mild urinary albumin < 30 mg/24 h; moderate urinary albumin with excretion between 30 and 300 mg/24 h; severe urinary albumin with excretion > 300 mg/24 h found in at least two consecutive measurements or with creatinine clearance < 50 mg/dl/24 h. With regard to the progression of renal damage, a significant increase in urinary albumin excretion or worsening of renal function indices were monitored. Pregestational arterial hypertension was defined as systolic blood pressure more than 140 mmHg and or diastolic blood pressure more than 90 mmHg or if a patient was taking antihypertensive drugs before pregnancy. Pregnancy-induced hypertension was diagnosed for systolic blood pressure values > 140 mmHg and diastolic > 90 mmHg after the 20th week of gestation.

As obstetric outcomes the loss of pregnancy before the 24th week of gestation, gestational hypertension, pre-eclampsia, caesarean section and preterm delivery were assessed. Preterm birth was defined as completion of birth before the 37th week. Perinatal/neonatal outcomes such as birth weight (grams and percentiles), birth length (cm and percentiles), foetal macrosomia defined as birth weight ≥ 90th percentile, hypoglycaemia, hypocalcaemia, jaundice and respiratory stress syndrome were extracted from the medical charts.

Total insulin requirement (IR) for each trimester and the change of IR from the end to the start of the trimester (∆_total_IR) were calculated.

### Assays

Blood glucose levels were measured by standard methods (Modular P800, Roche, Milan). HbA1c levels were determined by HPLC with an ion-exchange resin (Bio-Rad Laboratories, Milan, Italy).

### Statistical analysis

SPSS version 17 and MedCalc version 11.3 were used for data analysis. The differences between the two groups with *p*-values less than 0.05 were considered statistically significant.

Baseline characteristics were presented as mean ± SD for continuous variables; rates and proportions were calculated for categorical data. Normality of distribution for quantitative data was assessed by the Shapiro-Wilk test. To determine statistically significant differences between two groups (T1DM and T2DM) the unpaired Student’s t test for continuous variables (after testing for equality of variance: Levene test) and the chi square test and Fisher’s exact test (when appropriate) for categorical variables, were used.

ANOVA for repeated measures was used for comparison of the pregestational variables HbA1c, HbA1c at the first, second and third trimesters, pregestational BMI, maternal weight at early gestation and at delivery, total IR at early gestation and at delivery, ∆_total_IR in the first, second and third trimesters in the two groups of patients (T1DM and T2DM) after testing for equality of variance. The Fisher least significant difference post-hoc correction was applied if the variables had equal variances and the Dunnett post-hoc correction was applied if the variables did not have equal variances.

Crude odds ratios (OR) and their 95% confidence interval (CI) for the association of abortion and macrosomia with potential risk factors in pregestational diabetes mellitus were calculated by univariate analysis. Adjusted OR were calculated by stepwise logistic regression analysis to identify factors independently associated with the development of abortion and macrosomia. Only factors significantly associated with abortion and macrosomia by univariate analysis were included in the logistic regression analysis. The receiver operating characteristic (ROC) analysis was performed to investigate the diagnostic ability of significantly associated risk factors to predict abortion and macrosomia developments. The ROC curve was plotted as sensitivity versus 1-specificity. The area under the ROC curve (AUC) was estimated to measure the overall performance of the predictive factors for abortion and macrosomia. A *p* value of < 0.05 was considered statistically significant.

## Results

The clinical characteristics of pregnant women with pregestational T1DM and T2DM are shown in Table 1. Age at pregnancy was significantly lower (*p* < 0.001) and duration of diabetes was significantly longer in women with T1DM than in those with T2DM (*p* < 0.001). Pregestational body weight (*p* < 0.001), BMI (*p* < 0.001), and weight at delivery (*p* < 0.001) were significantly lower in women with T1DM than in those with T2DM (Table 1). However, the weight gain (∆_weight) was higher in women with T1DM than T2DM (*p* < 0.001).

**Table 1:** Maternal characteristics of women with type 1 diabetes mellitus (T1DM) and type 2 diabetes mellitus (T2DM)

	T1DM No. = 73	T2DM No. = 62	***p***
	***Mean ± SD***	***Mean ± SD***	
Duration of diabetes (years)	14.1 ± 8.05	4.11 ± 3.1	< 0.001
Age at pregnancy (years)	29.5 ± 5.42	33.7 ± 6.1	< 0.001
Pregestational BMI (Kg/m^2^)	21.8 ± 2.9	31.4 ± 6.7	< 0.001
Pregestational weight (Kg)	59.1 ± 8.8	82.2 ± 19.2	< 0.001
Weight at delivery (Kg)	72.5 ± 10.7	90.8 ± 20.1	< 0.001
∆_weight	13.5 ± 4.4	8.61 ± 6.8	< 0.001
Pregestational short-acting insulin dose (U/day)	28.7 ± 10.1	19.3 ± 10.9	0.001
Pregestational long-acting insulin dose (U/day)	17.7 ± 6.59	12.5 ± 8.53	0.016
Short-acting insulin dose at delivery (U/day)	45.4 ± 13.5	37.1 ± 20.3	0.037
Long-acting insulin dose at delivery (U/day)	21.8 ± 8.18	24.1 ± 10.1	0.353
	***Subjects (%)***	***Subjects (%)***	
Recurrent ketonuria	3 (4.2%)	4 (6.5%)	0.552
Pregestational diabetic retinopathy	16 (22.2%)	2 (3.2%)	0.016
Diabetic retinopathy progression	6 (8.3%)	1 (1.6%)	0.082
Pregestational diabetic nephropathy	10 (13.7%)	1 (1.6%)	0.018
Nephropathy progression	6 (8.3%)	5 (8.1%)	0.966
Pregestational arterial hypertension	3 (4.2%)	9 (14.5%)	0.037
History of recurrent miscarriages	7 (9.6%)	3 (4.8%)	0.290

Women with T1DM showed a higher prevalence of pregestational diabetic retinopathy (*p* = 0.016) and nephropathy (*p* = 0.018) (Table 1). By contrast, women with T2DM showed a higher prevalence of pregestational arterial hypertension (*p* = 0.037) than those with T1DM (Table 1).

**Fig. 1:**
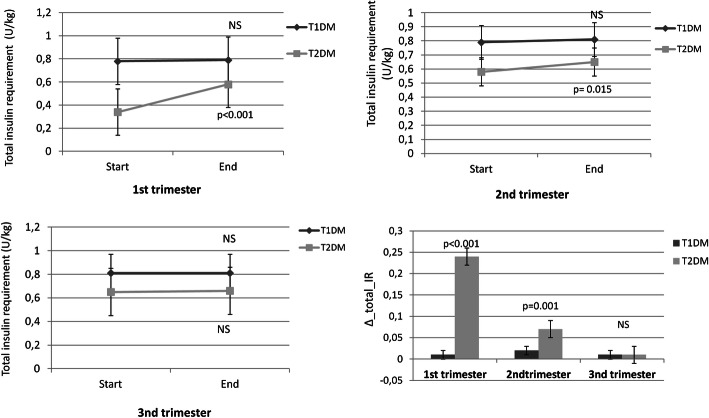
Total daily insulin requirements measured in units/kg during pregnancy (first, second and third trimesters) in type 1 and type 2 diabetes mellitus (T1DM and T2DM). Change (∆) from end to start of each trimester for insulin requirement (IR) in women with T1DM and T2DM

Patients with T1DM had significantly lower ∆_total_IR at the first (*p* < 0.001), second (*p* < 0.001) and third (*p* < 0.001) trimesters than T2DM (Fig. 1).

**Fig. 2:**
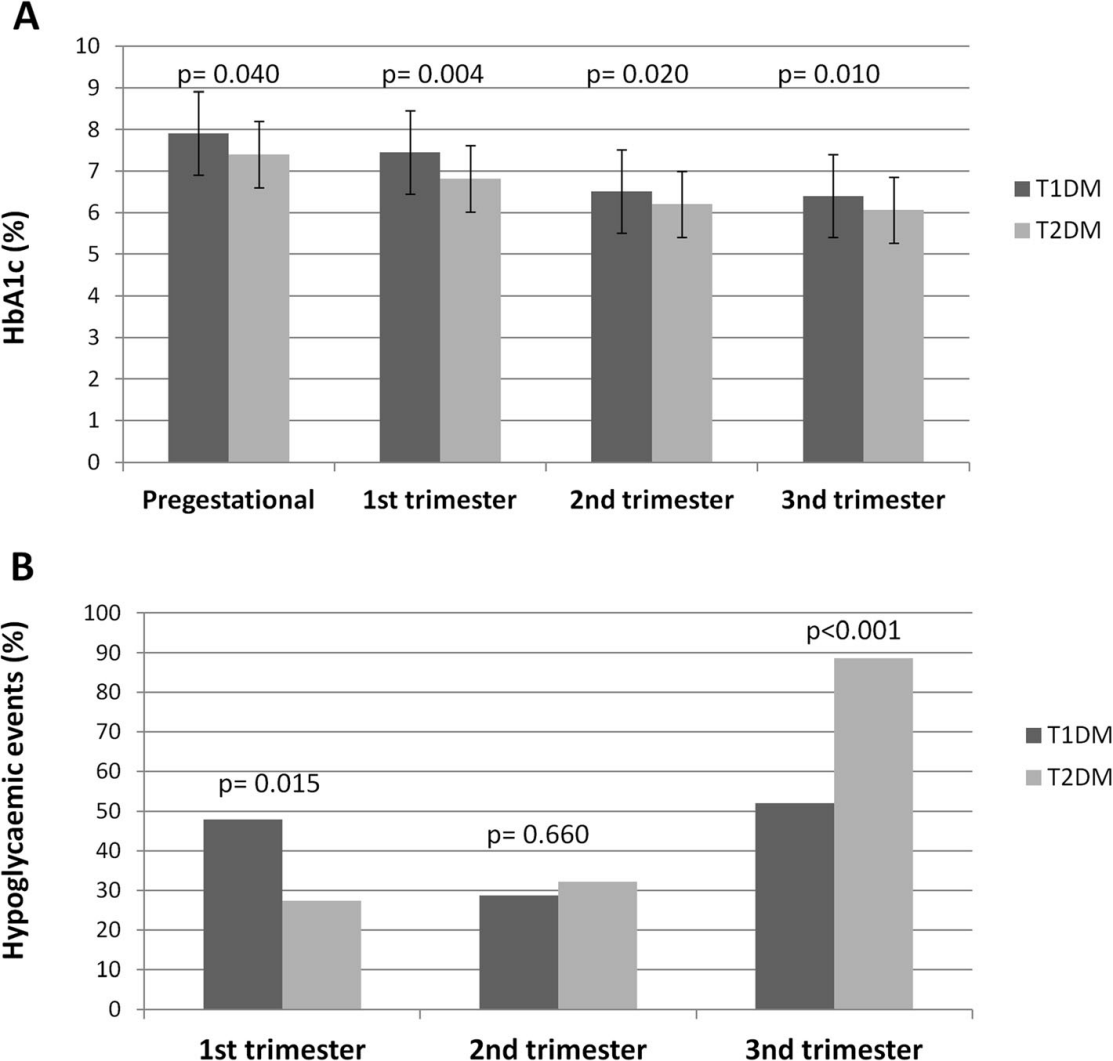
**a** HbA1c in preconception and during trimesters in pregnant women with T1DM (dark grey) vs. T2DM (light grey). Values are mean ± SD. Student’s test was used to evaluate the differences between the groups. **b** Number of pregnant women with T1DM and T2DM who experienced hypoglycaemic events during first, second and third trimester

In addition, patients with T1DM showed higher pregestational HbA1c (*p* = 0.040), HbA1c of first (*p* = 0.004), second (*p* = 0.020) and third (*p* = 0.010) pregnancy trimesters than those with T2DM (Fig. 2a). Women with T1DM showed a higher frequency of hypoglycaemic events in the first trimester (*p* = 0.015) (Fig. 2b) than T2DM, while women with T2DM showed a higher frequency of hypoglycaemic events in the third trimester than those with T1DM (*p* < 0.001) (Fig. 2b).

**Fig. 3:**
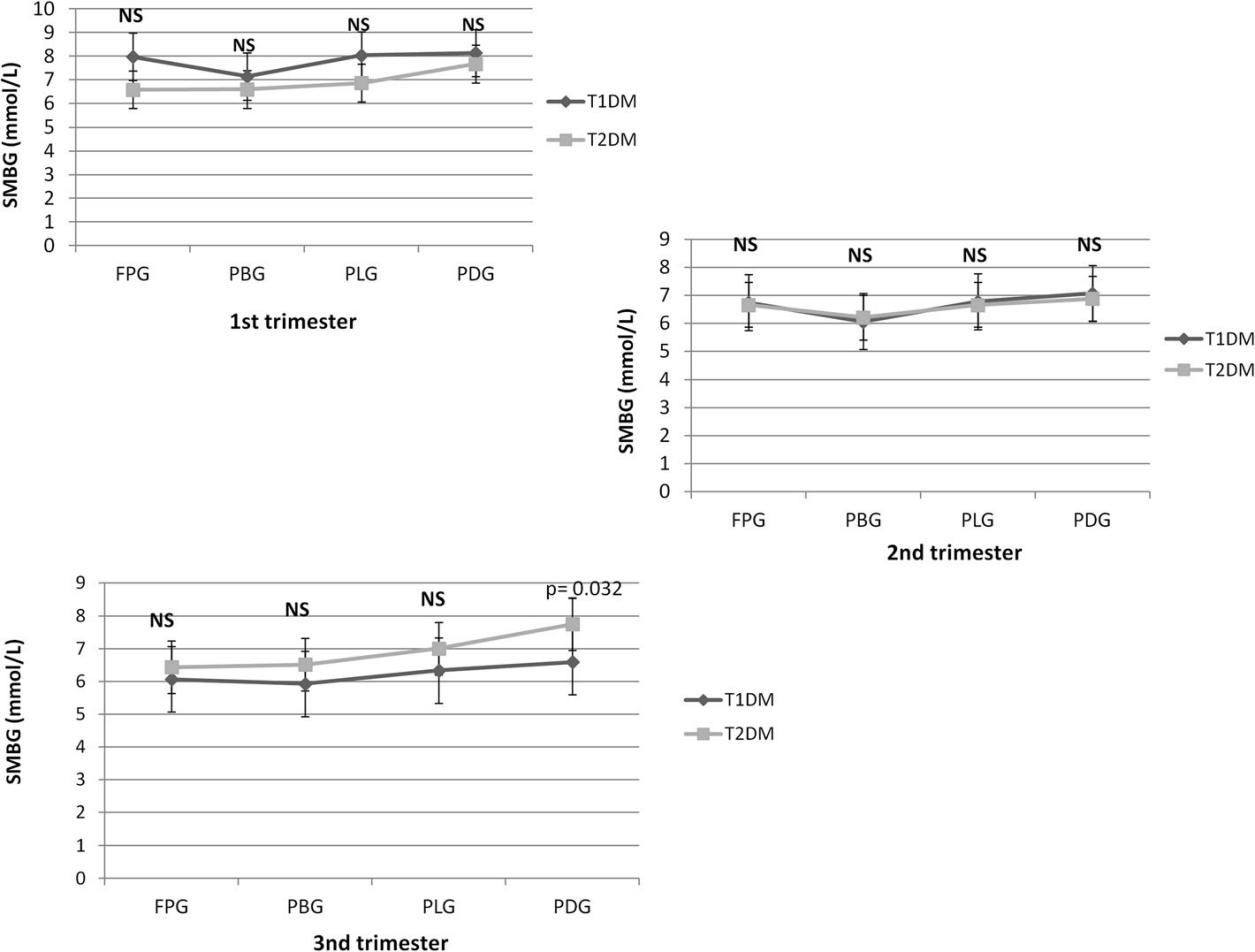
Average fasting and postprandial glycaemia obtained from SMBG in pregnant women with T1DM and T2DM. Data are presented as mean ± SD values. Student’s test was used to evaluate the differences between the groups. FPG: fasting plasma glucose. PBG: postprandial breakfast glucose. PLG: postprandial lunch glucose. PDG: postprandial dinner glucose. NS: not significant

No differences in average fasting and postprandial glycaemia obtained from SMBG were observed between the groups except for a higher post-dinner glycaemia level for women with T2DM than with T1DM (*p* = 0.022) (Fig. 3).

With regard to obstetric complications, women with T2DM had a higher frequency of spontaneous abortion (*p* = 0.037) than those with T1DM. By contrast, women with T1DM showed higher birth weight percentiles (*p* = 0.044) and a higher frequency of macrosomia (*p* = 0.005) than those with T2DM (Table 2).

**Table 2:** Obstetrical and neonatal/perinatal outcomes for women with type 1 diabetes mellitus (T1DM) and type 2 diabetes mellitus (T2DM)

	T1DM No. = 73	T2DM No. = 62	***p***
	***Subjects (%)***	***Subjects (%)***	
***Obstetrical complications***			
Gestational hypertension	15 (20.8%)	13 (21.0%)	0.977
Preeclampsia	9 (12.5%)	5 (8.2%)	0.418
Preterm delivery	23 (31.5%)	12 (19.1%)	0.102
Caesarean delivery	68 (93%)	50 (81.2%)	0.093
Abortion			
1st trimester	2 (2.6%)	8 (12.9%)	0.022
2nd trimester	0	2 (3.2%)	0.125
***Neonatal and perinatal outcomes***			
Large for gestational age (LGA)	36 (49.2%)	16 (25.8%)	0.005
Hypoglycaemia	24 (32.8%)	16 (25.8%)	0.376
Hypocalcemia	10 (13.6%)	4 (6.4%)	0.171
Jaundice	20 (27.4%)	13 (20.9%)	0.382
Respiratory distress syndrome	20 (27.4%)	12 (19.3%)	0.277
	***Mean ± SD***	***Mean ± SD***	
Birth weight (g)	3314 ± 650	3285 ± 700	0.803
Birth weight percentiles	72.6 ± 27.6	62.4 ± 30.7	0.044
Birth length (cm)	48.6 ± 3.3	48.6 ± 2.1	0.997
Birth length percentiles	60 ± 32.6	51.2 ± 28.6	0.100

At univariate analysis, women who had abortions had significantly higher values of pregestational BMI (*p* = 0.001), ∆_IR of first trimester (*p* = 0.003) and T2DM (*p* = 0.035) than women who did not abort (Table 3). In addition, women who had macrosomic infants had significantly higher weight gain during pregnancy (*p* < 0.001), ∆_IR of third trimester (*p* < 0.001), HbA1c of third trimester (*p* = 0.009) and T1DM (*p* = 0.012) (Table 4). At multivariate analysis, after stepwise selection in model 1 the significant variables that influenced the probability of having an abortion were the type of diabetes, with a higher risk in women with T2DM than T1DM (OR 3.03; *p* = 0.011), pregestational BMI (OR 2.06; *p* = 0.047) and the ∆_total_IR of the first trimester (OR 2.02; *p* < 0.001) (Table 5). In model 2 we observed that the macrosomia variable was influenced by the maternal weight gain with a risk of 2.36 for each kilogram of weight increase between early gestation and delivery (OR 2.36; *p* < 0.001) and T1DM (OR 2.65; *p* = 0.015) (Table 5). A ROC curve was constructed, and a prediction model was established with a moderately robust power (AUC = 0.73) to predict abortion and macrosomia in pregestational diabetic women.

**Table 3:** Obstetrical and neonatal/perinatal outcomes for women who had abortion or not

	***Abortion*** ***(N = 12)***	***No abortion*** ***(N = 123)***	
Duration of diabetes (years)	12.4 ± 9.76	14.6 ± 7.17	0.503
Age at pregnancy (years)	28.2 ± 6.01	30.4 ± 5.14	0.180
Pregestational BMI (kg/m^2^)	29.7 ± 7.76	25.3 ± 3.38	0.001
∆_insulin requirement 1st trimester	0.31 ± 0.12	0.23 ± 0.09	0.003
∆_insulin requirement 2nd trimester	0.15 ± 0.11	0.11 ± 0.05	0.346
Pregestational HbA1c	8.38 ± 1.77	7.88 ± 1.47	0.248
HbA1c 1st trimester	7.42 ± 1.16	7.51 ± 1.09	0.832
HbA1c 2nd trimester	6.72 ± 0.59	6.53 ± 0.79	0.476
FPG 1st trimester	6.78 ± 1.71	7.77 ± 3.47	0.143
PBG 1st trimester	7.21 ± 1.91	7.09 ± 2.26	0.861
PLG 1st trimester	6.56 ± 0.92	7.95 ± 2.41	0.093
PDG 1st trimester	7.73 ± 1.98	8.08 ± 2.98	0.650
FPG 2nd trimester	6.67 ± 1.97	6.69 ± 1.63	0.942
PBG 2nd trimester	6.42 ± 1.38	6.05 ± 1.82	0.453
PLG 2nd trimester	6.5 ± 1.12	6.71 ± 1.74	0.706
PDG 2nd trimester	7.01 ± 1.29	7.03 ± 1.81	0.944
	***Subjects (%)***	***Subjects (%)***	
Recurrent ketonuria	1 (8.3%)	6 (4.8%)	0.601
Pregestational diabetic retinopathy	2 (16.6%)	16 (13%)	0.727
Diabetic retinopathy progression	0	7 (8.8%)	0.501
Pregestational diabetic nephropathy	0	11 (8.9%)	0.282
Nephropathy progression	0	11 (8.9%)	0.282
Pregestational arterial hypertension	0	12 (9.7%)	0.260
History of recurrent miscarriages	0	10 (8.1%)	0.307
Gestational hypertension	2 (16.6%)	26 (21.1%)	0.714
Type of diabetes			
Diabetes type 1	3 (25%)	70 (56.9%)	0.035
Diabetes type 2	9 (75%)	53 (43.1%)	
Hypoglycaemic events 1st trimester	4 (28%)	47 (38%)	0.495
Hypoglycaemic events 2nd trimester	3 (20%)	31 (25%)	0.702

**Table 4:** Obstetrical and neonatal/perinatal outcomes for women who had or not macrosomic newborns

	***Macrosomia*** ***(N = 52)***	***No macrosomia*** ***(N = 83)***	
Duration of diabetes (years)	13.3 ± 8.58	14.6 ± 7.42	0.352
Age at pregnancy (years)	29.2 ± 6.01	30.4 ± 4.55	0.190
Pregestational BMI (kg/m^2^)	30.1 ± 3.76	28.9 ± 5.45	0.166
Weight gain (kg)	14.6 ± 3.58	9.2 ± 2.56	< 0.001
∆_insulin requirement 1st trimester	0.18 ± 0.15	0.16 ± 0.12	0.394
∆_insulin requirement 2nd trimester	0.11 ± 0.09	0.12 ± 0.08	0.501
∆_insulin requirement 3rd trimester	0.08 ± 0.03	0.05 ± 0.02	< 0.001
Pregestational HbA1c	7.78 ± 0.81	7.51 ± 0.76	0.056
HbA1c 1st trimester	7.48 ± 1.16	7.39 ± 0.98	0.629
HbA1c 2nd trimester	7.11 ± 0.68	6.73 ± 0.79	0.051
HbA1c 3rd trimester	7.01 ± 0.65	6.72 ± 0.57	0.015
FPG 1st trimester	6.24 ± 1.37	6.77 ± 2.48	0.160
PBG 1st trimester	6.74 ± 1.97	6.54 ± 2.26	0.601
PLG 1st trimester	6.89 ± 0.87	7.36 ± 2.37	0.172
PDG 1st trimester	7.93 ± 1.69	8.04 ± 2.94	0.806
FPG 2nd trimester	6.58 ± 1.76	6.73 ± 1.68	0.621
PBG 2nd trimester	6.56 ± 1.44	6.13 ± 1.75	0.140
PLG 2nd trimester	6.38 ± 1.47	6.92 ± 1.86	0.078
PDG 2nd trimester	7.13 ± 1.35	6.89 ± 2.01	0.448
FPG 3rd trimester	6.35 ± 1.87	6.23 ± 1.43	0.674
PBG 3rd trimester	6.45 ± 1.27	6.03 ± 1.47	0.091
PLG 3rd trimester	6.08 ± 1.39	6.34 ± 1.45	0.304
PDG 3rd trimester	6.94 ± 1.28	6.76 ± 1.78	0.527
	***Subjects (%)***	***Subjects (%)***	
Pregestational arterial hypertension	5 (9.6%)	7 (8.4%)	0.812
History of recurrent miscarriages	6 (11.5%)	4 (4.8%)	0.148
Gestational hypertension	12 (23.1%)	16 (19.3%)	0.597
Type of diabetes			
Diabetes type 1	35 (67.3%)	38 (45.7%)	0.012
Diabetes type 2	17 (32.7%)	45 (54.3%)	
Hypoglycaemic events 1st trimester	13 (25%)	25 (30%)	0.530
Hypoglycaemic events 2nd trimester	12 (23%)	25 (30%)	0.376
Hypoglycaemic events 3rd trimester	10 (20%)	20 (24%)	0.589

**Table 5:** Risk factors associated with abortion and macrosomia in patients with pregestational diabetes mellitus

Variable	Abortion *(N° = 12)*	No abortion *(N° = 123)*	Crude OR (95% CI)	Adjusted OR (95% CI)
**Pregestational BMI**				
≤ 27.5 kg/m^2^	4 (31.7%)	121 (98.3%)	1	1
> 27.5 kg/m^2^	8 (68.3%)	2 (1.7%)	3.76 (1.46–6.54)	2.06 (1.01–4.22)
**∆_insulin requirement 1st trimester**				
≤ 0.27	3 (25%)	101 (82.1%)	1	1
> 0.27	9 (75%)	22 (17.9%)	4.37 (1.61–11.8)	2.02 (1.08–4.02)
**Type 2 diabetes mellitus**				
No	3 (25%)	70 (56.9%)	1	1
Yes	9 (75%)	53 (43.1%)	11.5 (4.37–30.4)	3.03 (1.29–7.10)
	**Macrosomia** (*N*° = 52)	**No macrosomia** (*N*° = 83)		
**Weight gain**				
≤ 12.5 kg	10 (19.3%)	65 (78.3%)	1	1
> 12.5 kg	42 (80.7%)	18 (21.7%)	4.74 (1.86–12.1)	2.36 (1.44–3.87)
**∆_insulin requirement 3rd trimester**				
≤ 0.07	22 (42.3%)	60 (72.3%)	1	
> 0.07	30 (57.7%)	23 (27.7%)	1.25 (1–2.34)	
**HbA1c of 3rd trimester**				
≤ 6.88	24 (46.2%)	57 (68.7%)	1	
> 6.88	28 (53.8%)	26 (31.3%)	1.18 (1–2.09)	
**Type 1 diabetes mellitus**				
No	17 (32.7%)	45 (54.3%)	1	1
Yes	35 (67.3%)	38 (45.7%)	12.3 (3.84–39.3)	2.65 (1.14–3.98)

*Abbreviations*: *OR* odds ratio, *IC* confidence interval

## Discussion

Our data show that pregnant women with pregestational T2DM have a higher frequency of spontaneous abortion (within the 24th week of gestation) than women with pregestational T1DM and that it is correlated with the pregestational BMI and the ∆_total_IR of the first trimester. In addition, we found that women with pregestational T1DM have a higher frequency of perinatal complications, such as macrosomia, than women with T2DM and it is correlated with the higher weight gain during pregnancy.

Pregestational characteristics of women included in the current study, such as longer duration of diabetes, higher IR at early gestation, higher frequency of retinopathy and nephropathy in women with T1DM, and higher pregestational BMI and weight in women with T2DM are in line with those reported in other studies [8, 9].

In the current study women with T2DM had a higher incidence of spontaneous abortion. Pregestational BMI and ∆_total_IR of the first trimester and T2DM were risk factors of abortion. These findings suggest that obesity and consequent decrease of insulin sensitivity during the first trimester of pregnancy increase the risk of obstetrical complications. Women with T2DM require a much greater increase in insulin dose from the start to the end of each trimester with a progressive increase [10]. Although the increase in IR is presumably due to the effects of the placental hormones, some factors may have an influence in determining IR during pregnancy, such as pregestational BMI. An increase in adiposity is associated with higher production of pro-inflammatory cytokines and adipokines, which are responsible for the changes in insulin sensitivity [10].

Previous studies have shown that T2DM is associated with a higher incidence of early and late spontaneous abortion and in turn spontaneous abortion is associated with a high risk of developing T2DM [11]. Type 2 diabetes mellitus is associated with endothelial dysfunction and therefore with placenta abnormalities [12, 13]. The pregnancies of women with T2DM are known to be at higher risk of perinatal death, as well as congenital malformations, than those of women with T1DM [14]. In a study conducted by Clausen and colleagues [15], 61 women with T2DM were compared with 240 women with T1DM, demonstrating a 4-to-9 times higher incidence of foetal perinatal death in women of the first group compared to the second, although the latter had worse metabolic compensation. In a recent meta-analysis of 33 observational studies published in the last 20 years, women with T2DM had a higher incidence of perinatal death despite having a lower duration of diabetes, lower HbA1c values and lower rates of diabetic complications at the time of pregnancy than T1DM [14]. By contrast, McGrogan and colleagues [16] found a similar frequency of spontaneous abortion in women with T2DM and women with T1DM, although in any case the frequency was 20% higher than in the general population. In the current study, pregestational HbA1c was not associated with perinatal complications in women with T2DM, even though pregestational maternal glycaemic control is known to reduce perinatal complications in pregnant women with diabetes [17]. A recent meta-analysis has shown that despite less severe glycaemic disturbance, women with T2DM did not have better perinatal outcomes than those with T1DM, suggesting that factors other than glycaemic control also affect perinatal complications in women with T2DM [14]. Indeed, pregnancy-induced insulin resistance adds to the pre-existing insulin resistance, typical of T2DM, and the pre-existing pancreatic β-cell defect compromises the ability to enhance insulin secretion during pregnancy, leading to marked hyperglycaemia [12]. Pregnancy-induced metabolic changes in women with T2DM require more intensive monitoring and closer titration of treatment. Unlike normal pregnancies, which in the first trimester tend to have lower glucose values, in pregnant women with T2DM higher glucose spikes are generally observed and strict insulin therapy adjustment is required [12].

Taken together, the above findings regarding pregnancy in T2DM on a background of metabolic syndrome suggest that obesity and insulin resistance before and during the first trimester of pregnancy may greatly influence the risk of perinatal complications, more than glycaemic control [13, 16, 18].

With regard to women with T1DM, in the present study about 50% of them had perinatal/neonatal complications such as macrosomia and consequently higher birth weight percentiles, and this percentage was higher than in women with T2DM, in line with other studies [19]. We found maternal weight gain during pregnancy was a risk factor, rather than pregestational HbA1c. Similar results were obtained in a Danish observational study, carried out on a group of 115 women with T1DM, which demonstrated that weight gain was an independent risk factor for foetal overgrowth [20]. In addition, a retrospective analysis of pregnant women with T1DM showed that excess weight gain was correlated with high risk of macrosomia [21]. Other studies showed that, in addition to weight increase, HbA1c values also correlated with the risk of macrosomia. In a study conducted by Morrens and colleagues [22], out of 180 pregnant women with T1DM, there was an increased frequency of macrosomia in about 42.5% of cases, certainly a higher incidence than that of the general population, correlating this finding with both weight gain and HbA1c values in early gestation and at delivery. This result can be explained by longer duration of diabetes, greater glycaemic instability [22] and, according to other studies, greater weight of the placenta [23] in women with T1DM compared to women with T2DM. Also worthy of note is the need for greater surveillance for hypoglycaemias in macrosomic infants of women with T1DM given the 2.5-fold greater risk for these infants of hypoglycaemia. This factor can be considered a better predictor of neonatal hypoglycaemia, compared to maternal glycaemic control [24]. It has been suggested that glycaemic fluctuations and hypoglycaemia may influence the course of pregnancy in women with long-standing T1DM, but the effectiveness of different insulin treatments for glycaemic control and variability and hypoglycaemic episodes in pregnant women with T1DM has not been elucidated [25, 26].

The present study has some limitations. First, our study had a retrospective design. Second, the sample size was small. Third, data on placental histology are lacking and differences between placentas of women with T2DM and T1DM were not evaluated. Perhaps babies born to T1DM mothers are larger but in the same vein, babies born to mothers with T2DM may be smaller owing to endothelial dysfunction and placental pathology associated with T2DM. Fourth, data on Doppler changes on maternal and foetal vessels are not available. Because of these limitations, the results of the present study should be carefully interpreted. Nonetheless, we believe that our findings have an important implication for clinical management and treatment of pregnant women with diabetes. To confirm our findings, future studies with a prospective design and larger sample size including women with and without perinatal complications are warranted.

## Conclusions

Women with T2DM have a higher risk of spontaneous abortion than T1DM, due to pregestational BMI and ∆_total_IR in the first trimester, while women with T1DM have a higher risk of macrosomia than T2DM due to the weight gain throughout pregnancy. These findings are very interesting because highlight the association between obesity, gestational weight gain and pregestational diabetes and fetal outcomes and have an important implication for clinical management and treatment of pregnant women with diabetes.

## Data Availability

The datasets used and/or analysed during the current study are available from the corresponding author on reasonable request and are stored in the Google Drive of Endocrinology of University of Palermo.

## Abbreviations

T1DM: Type 1 diabetes mellitus
T2DM: Type 2 diabetes mellitus
BMI: Body mass index
HbA1c: Glycated hemoglobin
FPG: Mean fasting plasma glucose
PBG: Postprandial breakfast glucose
PLG: Postprandial lunch glucose
PDG: Postprandial dinner glucose
ROC: Receiver operating characteristic
AUC: Area under the ROC curve
SMBG: Self-monitoring blood glucose
IR: Insulin requirement
OR: Odds ratio
CI: Confidence interval
